# Feasibility of Strip Meniscometry for Tear Volume Evaluation in Lacrimal Passage Obstruction

**DOI:** 10.3390/diagnostics10040179

**Published:** 2020-03-25

**Authors:** Ikubun Osawa, Yuri Esaka, Takashi Kojima, Cem Simsek, Haruka Kudo, Murat Dogru

**Affiliations:** 1 Department of Ophthalmology, Japan Community Healthcare Organization Chukyo Hospital, Nagoya 457-8510, Japan; ikubun@chukyogroup.jp (I.O.); esaka@chukyogroup.jp (Y.E.); 2 Department of Ophthalmology, Keio University School of Medicine, Tokyo 160-8582, Japan; cemsimsek1@hotmail.com (C.S.); kudo_haruka@chukyogroup.jp (H.K.); muratodooru2005@yahoo.co.jp (M.D.); 3 Department of Ophthalmology, Mugla Sitki Kocman University School of Medicine, Mugla 48000, Turkey

**Keywords:** anterior segment optical coherence tomography, epiphora, lacrimal drainage system dysfunction, silicon tube intubation, strip meniscometry tube, tear meniscus

## Abstract

Strip meniscometry tube (SMTube) is useful for assessing tear volume in dry eye disease. We performed an in vitro and a clinical study to examine whether the SMTube can also be used to evaluate tear quantity in lacrimal passage obstruction (LPO). In vitro experiments showed that the SMTube value and the amount of water absorption were significantly correlated (*R*^2^ = 0.816, *p* = 0.0008), but the measured value peaked when 4 μL was absorbed. In the clinical study, 12 eyes of 9 LPO patients and 17 eyes of 17 healthy control subjects were included. The patients’ SMTube values were significantly higher than those of the control subjects (*p* < 0.05). The SMTube value significantly decreased after silicon tube intubation (*p* < 0.05). Tear meniscus height (TMH) and area (TMA), measured by anterior segment optical coherence tomography, exhibited a significant positive correlation with the SMTube value (TMH, *p* < 0.001, *R*^2^ = 0.396; TMA, *p* < 0.001, *R*^2^ = 0.366). Moreover, the SMTube value significantly correlated positively with the subjective symptom of epiphora (*p* = 0.012, *R*^2^ = 0.255). Thus, SMTube was useful for evaluating the tear volume and therapeutic effects in patients with LPO.

## 1. Introduction

Epiphora, the main symptom of lacrimal passage obstruction (LPO) is known to cause not only discomfort but also deterioration of visual acuity as the tear film thickness changes considerably with blinking [1]. LPO or stenosis is one of the causes of epiphora. Various surgeries have been performed for LPO, and it has been reported that improvement of epiphora by surgery significantly improves the quality of life [2].

In recent years, dacryoendoscopic surgery with silicone tube intubation has gained considerable popularity. Dacryoendoscopic surgery is performed not only for the treatment of common canalicular obstruction but also for nasolacrimal duct obstruction for which dacryocystorhinostomy (DCR) had been previously suggested.

It is reported that 75–90% of tears stored in the ocular surface are located in the tear meniscus [3]. For this reason, evaluating tear meniscus is an indicator of the tear volume retention at the ocular surface and also helps assess the therapeutic effects of lacrimal surgery. The Schirmer test and cotton thread test are the two widely used conventional tear function tests. However, these tests take some time to perform, and since a reflex secretion is induced, the test reproducibility is poor [4,5]. Other methods of tear volume assessment include the reflective meniscometry and the anterior segment optical coherence tomography (AS-OCT), which are minimally invasive methods for measuring tear volume without reflex tear secretion. AS-OCT is reportedly useful for quantitative evaluation of the tear volume, before and after surgery for LPO [6].

A strip meniscometry tube (SMTube) test involves a short examination time of 5 s and can be conducted without touching the ocular surface. It is possible to simply and quickly measure the tear volume, with minimal invasiveness, and can be performed anywhere without requiring any special instruments. Several studies have already shown that the SMTube is an effective test for evaluating dry eye disease [7,8,9,10]. However, to date, the effectiveness of using the SMTube for measuring tear volume in patients complaining of epiphora, and for quantitative evaluation of the therapeutic effect of lacrimal surgery, have not yet been thoroughly investigated.

In this study, we conducted in vitro experiments and preliminary clinical studies in patients who complained of epiphora due to LPO using the SMTube and examined whether the SMTube was feasible for evaluating the tear volume in patients with epiphora. Furthermore, we investigated whether this approach was useful for a quantitative evaluation of tear volume, before and after surgery for LPO.

## 2. Materials and Methods

### 2.1. In Vitro Experiment

An in vitro experiment was conducted to investigate whether the tear volume of patients with LPO could be measured using SMTube by employing the same methodology as in dry eye evaluation. Based on the average tear meniscus area (TMA = 0.10 mm^2^) and the previously reported palpebral fissure length (30 mm) of nasolacrimal duct obstruction patients [11], the amount of tear meniscus was assumed to be about 4 μL (3.88 μL to be exact), based on the following formula [12]:

Inferior Tear Meniscus Volume (mm^3^) = inferior palpebral fissure length (mm) × inferior TMA (mm^2^) × 1.294 = 30 mm × 0.10 mm^2^ × 1.294 = 3.882 mm^3^ = 3.882 μL

Based on this calculation, 0.5, 1, 1.5, 2, 2.5 3, 3.5, and 4 μL of saline solution were placed into the center of separate Petri dishes with a micropipette, and the saline was absorbed using a SMTube. The tip of the SMTube was immersed for 5 s to absorb the saline following a method previously reported in a dry eye study [9]. The saline absorption time and SMTube values were recorded for 0.5, 1, 1.5, 2, 2.5 3, 3.5, and 4 μL of each saline solution condition. The experiment was performed three times for each volume condition, and the average value was calculated.

### 2.2. Clinical Study

#### 2.2.1. Patients/Subjects

Retrospective pre-screening was conducted for patients who visited the Japan Community Healthcare Organization (JCHO) Chukyo Hospital from September 1, 2018 to May 1, 2019 with epiphora as the chief complaint. Among them, cases with eyelid abnormalities including ectropion, entropion, and conjunctival chalasis were excluded. Cases that had a diagnosis of LPO or stenosis and had a history of lacrimal passage surgery (dacryoendoscopic surgery with silicon tube intubation or dacryocystorhinostomy) were selected. Finally, 12 eyes of 9 patients were included in the study (2 male patients, 3 eyes; 6 female patients, 9 eyes; average age, 62 ± 13.4 years). For the age-matched control group, subjects with dry eyes (according to the Japan Dry Eye Society dry eye diagnostic criteria of 2016), epiphora (Munk score of ≥ 1 point), conjunctival chalasis, eyelid disease, and history of intraocular surgery were excluded. Finally, 17 eyes (6 male subjects, 6 eyes; 11 female subjects, 11 eyes; average age, 69.29 ± 7.16 years) were included in the control group.

This single-center study was approved by the institutional review board of JCHO Chukyo Hospital (2017023), and written informed consent was obtained from patients after providing a sufficient explanation of the study. The protocol of this study followed the tenets of the Declaration of Helsinki.

#### 2.2.2. SMTube Measurement

Under non-anesthetic conditions, the tip of the SMTube was immersed in the tear meniscus for 5 s under a slit-lamp microscope. The examination was performed while taking care to avoid the test paper touching the ocular surface. The measurement duration was strictly measured using a timer. The SMTube value (in millimeters) was measured from the wet length of the test paper. The SMTube examination was performed before the surgery, 3 weeks after the silicone tube intubation, and 4 weeks after the silicone tube removal. SMTube measurement was conducted by a single experienced examiner.

#### 2.2.3. Measurement of Tear Meniscus Using AS-OCT

For AS-OCT, the SS-1000 (CASIA, TOMEY, Nagoya, Japan) was used. A perpendicular line passing through the center of the cornea was taken as a section, and the tomography of the tear meniscus was obtained. Next, the distance from the contact point between the lower cornea and the tear meniscus to the lower eyelid and the contact point of the tear meniscus was measured as the tear meniscus height (TMH). The contour of the lower tear meniscus was traced, and the area inside the contour was calculated using a built-in program of the AS-OCT device. Since the light perpendicular to the anterior segment is refracted in the tear film and is not corrected on the OCT image, the measurement value was adjusted by dividing by the refractive index of water (1.343); this value was then defined as the tear meniscus area (TMA). The TMH and TMA were measured before the surgery, 3 weeks after the silicone tube intubation, and 4 weeks after the silicone tube removal. Measurement of tear meniscus using AS-OCT was conducted by a single experienced examiner.

#### 2.2.4. Subjective Symptoms Questionnaire

A subjective questionnaire about epiphora was administered using a visual analogue scale. Patients were asked to describe the symptoms of epiphora with 0 indicating no symptoms and 100 indicating intolerable symptoms. The questionnaire was conducted before the surgery and after the silicone tube removal.

#### 2.2.5. Dacryoendoscopic Surgery with Lacrimal Tube Intubation

The surgery began with the administration of nasal mucosal anesthesia and vasoconstriction using gauze soaked in a mixture of 4% lidocaine and epinephrine. Patients also received infratrochlear nerve-blocking anesthesia and subcutaneous infiltration anesthesia surrounding the punctum, with 1% lidocaine with 1:100,000 epinephrine. The superior and inferior puncta were dilated with a punctum dilator. The dacryoendoscope was inserted from each punctum; stenotic or occlusion sites were opened and enlarged by direct endoscopic probing. If stenosis or occlusion tissue could not be efficiently dealt with, sheath-guided endoscopic probing was performed [13]. Briefly, lacrimal passage stenosis or occlusion was released using a 20-G needle sheath on the tip of the endoscope. The sheath was left in the lacrimal pathway, xylocaine jelly was injected through the sheath to expand the lacrimal pathway, and then a silicone tube (LACRIFAST, KANEKA Medics, Tokyo, Japan) was inserted. Using a nasal endoscope, it was confirmed that both tips of the silicone tube had appeared in the opening of the nasolacrimal duct at the inferior nasal meatus. The surgery was performed by two experienced surgeons.

#### 2.2.6. Dacryocystorhinostomy Technique

Dacryocystorhinostomy was performed under local anesthesia in all cases. First, gauze soaked in a mixture of 1% epinephrine and oxybuprocaine hydrochloride was inserted into the ipsilateral nostril for mucosal anesthesia and vasoconstriction. Then, 1% lidocaine with 1:100,000 epinephrine was injected at the subcutaneous space of the medial canthal area. The skin was incised in a 2 cm arc between the medial canthus and the root of the nose. The orbicularis oculi muscle and the medial palpebral ligament were incised, and the ligament was detached from the periosteum of the frontal process of the maxillary. The bone around the lacrimal cavity was shaved to create a 1 cm^2^ bone window. The lacrimal sac and nasal mucosa were opened, and the anterior and posterior flaps were created. The posterior flaps of the lacrimal sac and nasal mucosa were sutured with 8-0 polypropylene. After dilatation of the superior and inferior lacrimal puncta, a lacrimal silicon tube (LCRIFAST EX, KANEKA Medics) was inserted. The tip of the tube was inserted into the nasal cavity. The anterior flaps were also sutured with 8-0 polypropylene. The wound was closed with 7.0 nylon in the order of internal horn ligament, the muscle layer, the subcutaneous tissue, and finally the skin. The surgery was performed by three experienced surgeons.

### 2.3. Statistical Analyses

The Mann–Whitney U test was used to compare the values obtained between the healthy controls and patients. The Friedman test was used to evaluate the time-course changes in the SMTube value, TMH, and TMA. Wilcoxon’s signed-rank test was used to evaluate the questionnaire score over time. Spearman’s rank correlation coefficient was used to analyze the correlation between the questionnaire score and the SMTube value, TMH, and TMA. All statistical analyses were performed using SPSS (ver. 9, IBM, Armonk, NY, USA). A *p* value of less than 5% was considered to be statistically significant.

## 3. Results

### 3.1. In Vitro Experiment

**Figure 1:**
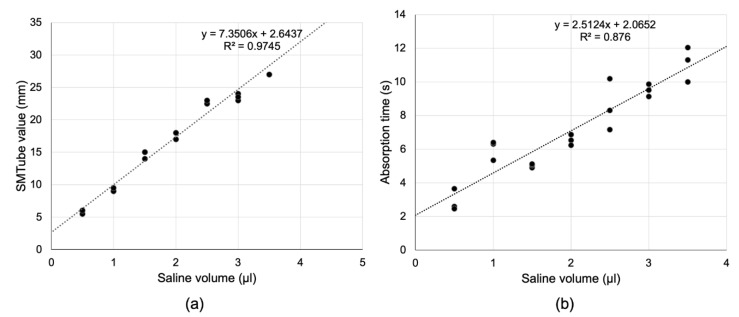
Results of in vitro experiments. (**a**) The strip meniscometry tube (SMTube) value correlated significantly with the saline volume. (**b**) Similarly, the absorption time correlated significantly with the saline volume.

Figure 1a shows the relationship between the saline volume and the absorption time in the in vitro experiment. There was a significant correlation between the saline volume and the absorption time (Figure 1a, *R*^2^ = 0.816, *p* = 0.0008). Figure 1b shows the relationship between the saline volume and the SMTube value. There was a significant positive correlation between the saline volume and the SMTube value (Figure 1b, *R*^2^ = 0.984, *p* < 0.0001). When more than 4 μL of saline was absorbed by the SMTube, the corresponding value was excluded as it exceeded the upper limit of measurement of the SMTube (27 mm).

### 3.2. Clinical Study

#### 3.2.1. Comparison between Control Subjects and Patients

**Figure 2:**
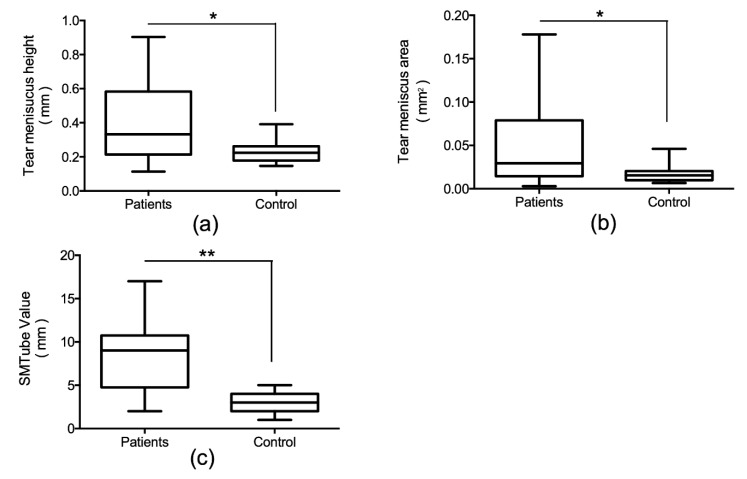
Comparison of tear meniscus parameters measured by optical coherence tomography and SMTube value between the lacrimal passage obstruction patients and control subjects. Tear meniscus height (**a**) and area (**b**) in patients with lacrimal passage obstruction was significantly greater than that in the control subjects. SMTube value in the patients with lacrimal passage obstruction was also significantly greater than that in the control subjects (**c**). * *p* < 0.05. ** *p* < 0.001.

Figure 2 shows the comparison of TMH, TMA, and SMTube values of patients with lacrimal passage obstruction and the control subjects. The TMH, TMA, and SMTube values in patients with lacrimal passage obstruction were significantly higher than that in the control subjects (TMH, *p* = 0.046; TMA, *p* = 0.028; SMTube value, *p* = 0.0002).

#### 3.2.2. Time-Course Changes of TMH, TMA, and SMTube Values before and after Treatment in Patients with Lacrimal Passage Obstruction

**Figure 3:**
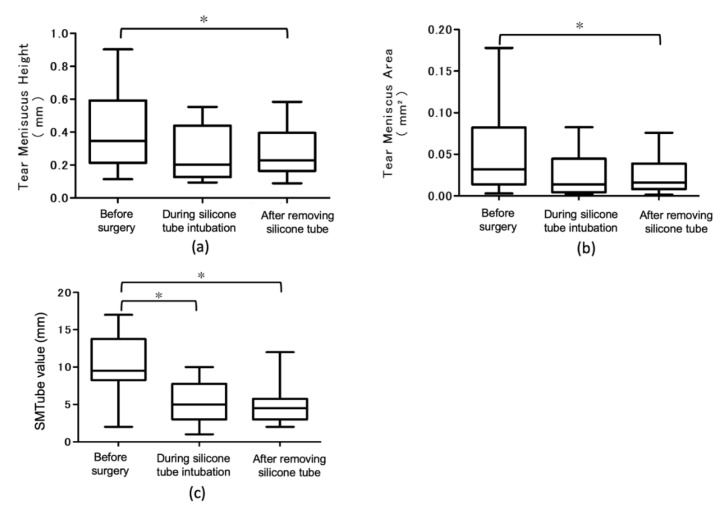
Time-course changes of tear meniscus height, tear meniscus area, and strip meniscometry value before surgery, during the silicone tube intubation, after removing the silicone tube. (**a**) Tear meniscus height decreased significantly after removing the silicone tube, compared to before the surgery. (**b**) The tear meniscus area decreased significantly after removing the silicone tube, compared to before the surgery. (**c**) The SMTube value decreased significantly after removing the silicone tube compared to before the surgery and during the silicone tube intubation. * *p* < 0.05.

Figure 3 shows changes in TMH, TMA, and SMTube values before, during the silicone tube intubation, and after the removal of the silicone tube. TMH and TMA were significantly decreased after the removal of the silicone tube (*p* < 0.05). The SMTube value decreased significantly during the silicone tube intubation and after the removal of the silicone tube (*p* < 0.05).

#### 3.2.3. Correlation between the Anterior Segment OCT Parameters and SMTube Values

**Figure 4:**
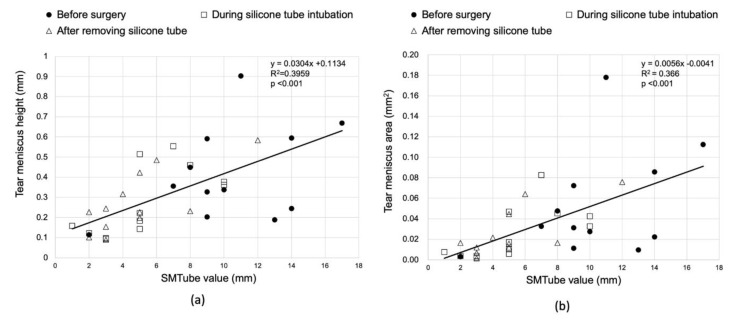
Correlation between the anterior segment optical coherence tomography (OCT) parameters and SMTube value. (**a**) The tear meniscus height significantly correlated with the SMTube value. (**b**) The tear meniscus area also correlated significantly with the SMTube value.

The relationship between the TMH and SMTube value before the surgery, during the silicon tube intubation, and after the removal of the silicon tube is shown in the scatter diagram in Figure 4. The TMH and SMTube values exhibited a significant positive correlation (Figure 4a, *p* < 0.001, *R*^2^ = 0.396). Similarly, there was a significant positive correlation between the TMA and SMTube values (Figure 4b, *p* < 0.001, *R*^2^ = 0.366).

#### 3.2.4. Correlation between the Patient Questionnaire and Anterior Segment OCT Parameters/SMTube Value

Epiphora symptoms before the surgery were evaluated by visual analog scale (VAS) and the correlations with AS-OCT parameters/SMTube values were examined. TMH and TMA values showed no significant correlation with the VAS scores (TMH, *p* = 0.012; TMA, *p* = 0.091), while the SMTube value showed a significant correlation with the VAS scores (*p* = 0.012).

## 4. Discussion

The SMTube was developed for diagnosing dry eye and reportedly allows measurement of the tear meniscus in 5 s. In the current study, we evaluated whether the SMTube could be used in patients with a high tear meniscus due to LPO.

In the in vitro experiments, it took longer than 5 s to absorb 1 μL of saline. In addition, it took 6.5 and 9.5 s to absorb 2 and 3 μL of saline, respectively. There was a positive correlation between the amount of saline absorbed and the SMTube value; however, when 4 μL of saline was absorbed, the measured value reached the upper limit of measurement by the SMTube (27 mm). The average tear meniscus area of patients with LPO obtained in this study was 0.05 ± 0.05 mm^2^. Assuming a horizontal inferior eyelid length of 30 mm, the lower tear meniscus volume was estimated to be 1.94 ± 1.94 μL. When the obtained tear volume was applied to the in vitro experimental results, the 5 s measurement time was considered to be insufficient. In addition, depending on the case, it was assumed that the upper limit of SMTube measurement could be reached. In reality, however, the 5 s measurements correlated well with the tear meniscus height measured by the AS-OCT, and the upper limit of the SMTube measurement value was not exceeded.

In clinical studies, it is possible that not all the tears that accumulated in the lower tear meniscus could be absorbed. The reason for the high correlation with the AS-OCT parameters may be the following: the radius of curvature of the tear meniscus reflects the amount of tear fluid [14]. The SMTube reduced the tear meniscus volume and decreased the tear meniscus radius. Based on the Laplace equation reflecting the relationship between the surface tension and the radius of curvature, negative pressure at the tear meniscus increases so that the tears stay on the meniscus. Due to this effect, it is possible that not all the tears at the meniscus could be absorbed. From this point of view, it was considered that the SMTube value reflected the water content of the tear meniscus even if all the fluid at the entire tear meniscus could not be absorbed.

In the comparison of the age-matched control group and LPO group before the surgery, the LPO group showed significantly higher TMH and TMA values than those in the control group. Similarly, the SMTube value in the LPO group was significantly higher than that in the control group. The SMTube test could, therefore, be useful for diagnosing LPO if the cut-off values are determined in a future study that includes a large number of cases. In the LPO group, the SMTube value was significantly correlated positively with both TMH and TMA. This indicated that the SMTube test results reflected the retained tear volume in the lower tear meniscus.

In addition, we evaluated the tear volume with AS-OCT and SMTube testing before and after the lacrimal passage surgery. Both TMH and TMA values measured by AS-OCT were significantly decreased after the silicone tube removal as compared to the preoperative values. Similarly, the SMTube values decreased significantly both during the silicone tube insertion and after the tube removal, as compared to before the surgery. These findings suggest that SMTube can be effective for judging the effect of lacrimal passage treatment.

The VAS symptom score for the degree of epiphora improved significantly after the surgery. Interestingly, a correlation was observed between the VAS score and the SMTube values, but no correlation was observed between the VAS score and the TMH or TMA. When we observed patients with epiphora using slit-lamp microscopy, tear accumulation was often found not only in the tear meniscus but also near the Marx line. The SMTube test paper gets in contact with the tear meniscus and absorbs the tears. In this process, the SMTube may also absorb the tear fluid stored in areas other than in the tear meniscus. This suggests that SMTube values may reflect subjective symptoms more efficiently than AS-OCT. While epiphora is associated with various symptoms such as blurred vision and ocular discomfort, only the degree of epiphora was evaluated as a questionnaire item in this study. In the future, it will be necessary to implement a multifaceted questionnaire including other symptoms and examine the correlation of these values with each test result in further detail.

Tear meniscus measurement is an important indicator for diagnosing LPO as well as dry eye. Ibrahim et al. reported a strong correlation between TMH and strip meniscometry values measured in AS-OCT in dry eye patients [15]. In our study, the 5 s SMTube measurement value correlated with TMH and TMA from AS-OCT examinations; however, the SMTube test was performed only once. The disadvantage is that it cannot be examined multiple times on the same day in contrast to the AS-OCT test. Although it has been reported that the SMTube test has good reproducibility [8], there are no reports in patients with epiphora. In the future, reproducibility needs to be further investigated in this subset of subjects.

Tear volume measurement by AS-OCT is a non-invasive, accurate, and reproducible method for the quantitative evaluation of the tear meniscus. Meanwhile, the SMTube is an examination instrument that can be used in any facility, without any special equipment. In addition, it can be measured without ophthalmic anesthetics thus avoiding a possible reflex tear secretion. However, as a disadvantage, the examiner needs to be trained in the examination procedure to ensure that the test paper does not touch the ocular surface. In a recent study, subjects assessed their diurnal tear meniscus variation by a self-examination protocol, suggesting that the SMTube could be useful for evaluating the diurnal variation of the tear meniscus in patients with epiphora in future.

One of the limitations of this study is that the clinical study was a preliminary investigation for evaluating the feasibility of using the SMTube test in patients with epiphora. For this reason, we examined the usefulness of SMTube in a small number of patients. The clinical study was also conducted at a single institution. Further multicenter studies with a larger number of patients are required. The dacryoendoscopic surgery and dacryocystorhinostomy were performed by two and three surgeons, respectively. Measurement of tear volume using the SMTube was also performed by one skilled examiner. It is, therefore, necessary to examine whether there was no influence or bias of the surgeon’s skill and the surgical method, by using multiple examiners.

In this study, the SMTube was effective as a tear volume evaluation test for patients with LPO, and the effectiveness of the quantitative evaluation of lacrimal surgery was demonstrated. This test is advantageous in terms of saving time and cost, without requiring any specialized equipment. Diverse diseases can cause epiphora, for instance, ocular surface diseases such as conjunctival chalasis and functional epiphora, which is thought to be due to lacrimal pump dysfunction. In the future, the effectiveness of the SMTube test should be further evaluated in patients with epiphora related to such causes.
